# Relationship between distribution and severity of non-perfusion and cytokine levels and macular thickness in branch retinal vein occlusion

**DOI:** 10.1038/s41598-020-79522-5

**Published:** 2021-01-11

**Authors:** Gahyung Ryu, Donghyoun Noh, Jano van Hemert, SriniVas R. Sadda, Min Sagong

**Affiliations:** 1 Department of Ophthalmology, Yeungnam University College of Medicine, #170 Hyunchungro, Nam-gu, Daegu, 42415 South Korea; 2 Yeungnam Eye Center, Yeungnam University Hospital, Daegu, South Korea; 3 Good Doctors Eye Hospital, Ulsan, South Korea; 4 Optos PLC, Dunfermline, UK; 5 Doheny Image Reading Center, Doheny Eye Institute, Los Angeles, CA USA; 6 Department of Ophthalmology, David Geffen School of Medicine At UCLA, Los Angeles, CA USA

**Keywords:** Cytokines, Retina, Retinal diseases, Medical research, Imaging and sensing, Angiogenesis, Vascular diseases

## Abstract

We aimed to investigate the relationship between non-perfusion on ultra-widefield angiography (UWF FA) and aqueous cytokine levels and central macular thickness (CMT) in eyes with branch retinal vein occlusion (BRVO). Thirty-five eyes with treatment-naïve BRVO were included. Non-perfusion area (NPA) for partial and complete ischemia was manually segmented and the ischemic index (ISI) for each was calculated using stereographically projected UWF FA for four different retinal zones. Partial and complete ischemia had different regional predominance. Partial ischemia was predominant in the posterior regions, while complete ischemia was predominant in the periphery. And partial ischemic area, located posterior to far periphery, showed significant correlation with central macular thickness and concentrations of angiogenic and inflammatory cytokines, while complete ischemic area showed no correlation with any of the parameters. Taken together, partial but not complete ischemia, particularly in the more posterior retina, was associated with higher cytokine levels and more severe macular edema in eyes with BRVO. These findings would help us to better understand the different clinical significance of ischemia in BRVO depending on the severity and regional distribution.

## Introduction

Branch retinal vein occlusion (BRVO) is a very common retinal vascular disorder, and can result in retinal hemorrhage, edema, and ischemia^1^. Retinal ischemia is accompanied by increased expression of vascular endothelial growth factor (VEGF) and inflammatory cytokines, resulting in breakdown of the blood-retina barrier and increased vascular permeability, which leads to development of macular edema (ME)^1^.

Several studies have reported a relationship between the severity of retinal ischemia and concentrations of cytokines in BRVO^2,3^. Kaneda et al. found that pretreatment non-perfusion area (NPA) showed significant correlation with the aqueous concentrations of IL-2, IL-6, IL-8, and MCP-1^2^. Noma et al. reported that vitreous fluid levels of VEGF, sICAM-1, IL-6, MCP-1, and PTX3 showed significant correlation with NPA in BRVO^3^. The extent of retinal ischemia and concentrations of cytokines in patients with BRVO has also been correlated with the clinical course and response to the treatment^2–6^, suggesting a potential prognostic role for these biomarkers.

However, the definitions of perfusion status in previous studies were variable and did not clearly define capillary non-perfusion, resulting in conflicting reports about the perfusion status around the macula^7–9^. Accordingly, Sakimoto et al. developed a new grading system that divides the perfusion status into three grades: full perfusion, partial perfusion, and complete obstruction^10^, but due to the technological limitations of standard fluorescein angiography (FA), the relationship between peripheral retinal perfusion and ME was not able to be evaluated. The advent of ultra-widefield (UWF) imaging systems has allowed more than 80% of the retinal surface to be viewed while displaying all vessels in the same angiographic phase. However, initially studies using UWF quantified NPA by expressing the number of pixels rather than as precise areas^11,12^. Advances in UWF software has now enabled the real physical area of the lesions on the retina in mm^2^ to be calculated by accounting for the non-linear distortion^13,14^. With these software tools, the size of a pixel can be defined by its location in the image and calculated using spherical trigonometry after it is projected back onto a sphere.

In the present study, we classified ischemic status into complete and partial ischemia and quantified the precise size of NPA in different retinal zones using stereographically projected UWF FA in eyes of patients with ME secondary to BRVO. We also evaluated the relationship between the extent of non-perfusion in different regions and the aqueous levels of various cytokines, visual acuity, and macular thickness.

## Results

### Demographic features

Thirty-five eyes of 35 patients with treatment-naïve BRVO were included in the study. Mean duration of disease was 4.9 ± 5.8 months. Mean age was 63.7 ± 12.6 years and 15 patients (42.9%) were male. Mean BCVA was 0.55 ± 0.42 logMAR and mean spherical equivalent was 0.09 ± 2.01 diopters with a mean central macular thickness (CMT) of 567.18 ± 166.83 µm. The 22 patients (36.4% male) who were scheduled to have cataract surgery who served as the control group had a mean age of 64.6 ± 8.7 years and mean spherical equivalent of − 0.41 ± 1.61 diopters. The two groups did not differ significantly in terms of age, gender, and spherical equivalent (*P* = 0.954, *P* = 0.783, and *P* = 0.092, respectively) (Table 1). However, patients with BRVO showed significantly higher concentrations of Ang-2, MCP-1, IL-8, IL-6, PlGF, and VEGF-A than the age-matched control group (*P* = 0.028, *P* = 0.005, *P* < 0.001, *P* = 0.006, *P* < 0.001, and *P* < 0.001, respectively). There was no statistically significant difference in Ang-1 or PDGF-AA levels between the two groups (*P* = 0.207) (Table 1).

**Table 1:** Demographic features of patients with retinal vein occlusion and control group.

Demographic features	BRVO (n = 35)	Control (n = 22)	*P*-value
Age, years; mean ± SD	63.7 ± 12.6	64.6 ± 8.7	0.954*
Sex, n (%)			0.783^†^
Male	15 (42.9)	8 (36.4)	
Female	20 (57.1)	14 (63.6)	
Disease duration, months; mean ± SD	4.9 ± 5.8		
Spherical equivalent, diopters; mean ± SD	0.09 ± 2.01	− 0.41 ± 1.61	0.092*
BCVA, logMAR; mean ± SD	0.55 ± 0.42	0.36 ± 0.41	0.028*
CMT, µm; mean ± SD	567.18 ± 166.83	265.77 ± 18.69	< 0.001*
Baseline measurements using UWF FA; mean ± SD			
NPA, mm^2^	208.28 ± 76.15		
TRA, mm^2^	715.86 ± 51.82		
ISI	0.28 ± 0.10		
Baseline levels of cytokines, pg/ml; mean ± SD			
Ang-1	47.51 ± 56.37	35.08 ± 36.59	0.426*
Ang-2	77.42 ± 98.90	27.53 ± 36.88	0.028*
MCP-1	919.53 ± 381.07	646.55 ± 247.83	0.005*
IL-8	42.76 ± 36.26	12.05 ± 10.23	< 0.001*
IL-6	46.19 ± 191.34	3.98 ± 5.74	0.005*
PDGF-AA	22.48 ± 8.49	24.94 ± 7.92	0.207*
PlGF	1.63 ± 1.25	0.71 ± 0.50	< 0.001*
VEGF-A	122.21 ± 75.98	57.64 ± 22.48	< 0.001*

BCVA: best-corrected visual acuity, BRVO: branch retinal vein occlusion, CMT: central macular thickness, ISI: ischemic index, logMAR: logarithm of the minimum angle of resolution, NPA: non-perfusion area, SD: standard deviation, TRA: total visible retinal area, UWF FA: ultra-widefield fluorescein angiography.

Numerical data are presented as mean ± standard deviation.

*Mann–Whitney U test, ^†^Chi square test.

### Distribution of ischemic area within different retinal zones

For the retina as a whole, total NPA in patients with BRVO was 208.28 ± 76.12 mm^2^, consisting of 144.52 ± 60.63 mm^2^ of partial ischemia and 63.76 ± 53.05 mm^2^ of complete ischemia (Table 2). The proportions of partial ischemia to total ischemia in peri-macular region (PMR), near-peripheral region (NPR), mid-peripheral region (MPR), and far-peripheral region (FPR) were 83.6%, 78.8%, 63.8%, and 58.0%, respectively (*P* < 0.001 for all inter-regional comparisons). Analogously, the proportions of complete ischemia to total ischemia were higher in the periphery than in the posterior zone (16.4%, 21.2%, 36.2%, and 42.0% for PMR, NPR, MPR, and FPR, respectively; *P* < 0.001 for all inter-regional comparisons).

**Table 2:** Distribution of ischemic area within different retinal zones.

	NPA, mm^2^ (%)	*P*-value	ISI	*P*-value
Total ischemia	208.28 ± 76.12 (100)		0.28 ± 0.10	
PMR	10.13 ± 4.38 (4.9)	< 0.001	0.38 ± 0.16^c^	0.007
NPR	78.57 ± 33.00 (37.8)		0.30 ± 0.12^b^	
MPR	81.28 ± 35.91 (39.1)		0.27 ± 0.12^a^	
FPR	38.11 ± 25.03 (18.3)		0.30 ± 0.15^ab^	
Partial ischemia	144.52 ± 60.63 (100)		0.20 ± 0.08	
PMR	8.46 ± 4.53 (5.9)	< 0.001	0.31 ± 0.17^c^	< 0.001
NPR	61.90 ± 28.95 (42.8)		0.23 ± 0.11^b^	
MPR	51.89 ± 30.65 (35.9)		0.17 ± 0.10^a^	
FPR	22.11 ± 21.20 (15.3)		0.17 ± 0.16^ab^	
Complete ischemia	63.76 ± 53.05 (100)		0.09 ± 0.08	
PMR	1.67 ± 3.45 (2.6)	< 0.001	0.06 ± 0.13^a^	< 0.001
NPR	16.67 ± 23.46 (26.1)		0.06 ± 0.09^a^	
MPR	29.39 ± 26.19 (46.1)		0.10 ± 0.09^b^	
FPR	16.00 ± 13.31 (25.1)		0.13 ± 0.10	

FPR: far-peripheral region, ISI: ischemic index, MPR: mid-peripheral region, NPA: non-perfusion area, NPR: near-peripheral region, PMR: peri-macular region.

Numerical data are presented as mean ± standard deviation. *P*-values were calculated using Friedman test. The letters a, b, c denote statistical significance between each group of post-hoc comparison: groups sharing the same letter do not significantly differ by Wilcoxon signed-rank test.

Among the BRVO eyes, total ischemic index (ISI) was 0.28 ± 0.10, partial ISI was 0.20 ± 0.08, and complete ISI was 0.09 ± 0.08. As the area of the various zones differs (with MPR occupying the largest area), comparative analysis based on ISI is of particular relevance. The total ISI values were the highest in PMR followed by NPR, FPR, and MPR (*P* = 0.007). The differences in ISI among the retinal zones were also significant in both cases of partial and complete ischemia (*P* < 0.001 for both) (Table 2). A high level of agreement was observed between graders for the ISI and NPA (kappa 0.88–0.94).

### Correlation of ischemia in different retinal zones with cytokines, BCVA, and CMT

Total NPA from the whole retina showed significant positive correlations with aqueous levels of IL-8 (r = 0.385, *P* = 0.023), PlGF (r = 0.364, *P* = 0.031), and VEGF-A (r = 0.418, *P* = 0.012). On the regional analysis, BCVA showed significant correlation with total NPA only for the PMR. CMT showed correlation with the total ischemia in PMR and NPR. And aqueous levels of Ang-2, MCP-1, IL-8, PlGF and VEGF-A were correlated with total NPA in NPR and MPR. Levels of PDGF-AA showed correlation with total NPA only in the PMR. The correlations were similar when assessed by ISI (Table 3).

**Table 3:** Correlation of total ischemia in different retinal zones with visual acuity, central macular thickness, and levels of cytokines.

	BCVA		CMT		Ang-1		Ang-2		MCP-1		IL-8		IL-6		PDGF-AA		PlGF		VEGF-A	
	R	*P*	R	*P*	R	*P*	R	*P*	R	*P*	R	*P*	R	*P*	R	*P*	R	*P*	R	*P*
NPA	0.042	0.821	0.224	0.204	0.266	0.122	0.298	0.082	0.325	0.056	0.385	0.023*	0.231	0.182	0.108	0.537	0.364	0.031*	0.418	0.012*
PMR	0.389	0.028*	0.450	0.008*	− 0.111	0.524	0.066	0.708	0.114	0.515	0.133	0.447	− 0.122	0.487	0.336	0.048*	0.251	0.146	0.123	0.483
NPR	0.155	0.396	0.361	0.036*	0.124	0.479	0.383	0.023*	0.377	0.026*	0.461	0.005*	0.260	0.131	0.060	0.732	0.471	0.006*	0.454	0.006*
MPR	0.029	0.873	0.182	0.302	0.308	0.072	0.339	0.046*	0.366	0.031*	0.366	0.031*	0.263	0.127	0.118	0.498	0.365	0.031*	0.370	0.029*
FPR	− 0.312	0.082	− 0.213	0.226	0.248	0.151	− 0.124	0.477	− 0.101	0.564	− 0.143	0.412	− 0.061	0.727	0.012	0.947	− 0.186	0.284	− 0.031	0.862
ISI	0.061	0.740	0.297	0.088	0.186	0.284	0.364	0.031*	0.350	0.039*	0.451	0.007*	0.207	0.232	0.166	0.341	0.450	0.007*	0.445	0.007*
PMR	0.443	0.011*	0.383	0.025*	− 0.083	0.634	0.091	0.604	0.131	0.453	0.069	0.692	− 0.146	0.404	0.290	0.091	0.229	0.186	0.114	0.513
NPR	0.156	0.395	0.371	0.031*	0.098	0.574	0.293	0.088	0.330	0.053	0.461	0.005*	0.209	0.228	0.052	0.768	0.441	0.008*	0.427	0.010*
MPR	− 0.034	0.854	0.145	0.414	0.220	0.205	0.326	0.056	0.314	0.066	0.384	0.023*	0.190	0.275	0.114	0.515	0.375	0.027*	0.343	0.043*
FPR	− 0.283	0.117	− 0.127	0.476	− 0.055	0.752	0.008	0.962	0.012	0.945	0.049	0.781	− 0.094	0.592	0.083	0.634	0.055	0.754	0.087	0.620

BCVA: best-corrected visual acuity, CMT: central macular thickness, FPR: far-peripheral region; ISI: ischemic index, MPR: mid-peripheral region, NPA: non-perfusion area, NPR: near-peripheral region, PMR: peri-macular region, R: Spearman’s correlation coefficient.

*P*-values were calculated using Spearman’s correlation.

*P-value < 0.05.

Partial NPA from the whole retina showed significant correlation with more parameters including Ang-1, Ang-2, MCP-1, and IL-6 than total NPA. On the regional analysis, CMT showed significant correlation with the partial NPA in PMR and NPR. And aqueous levels of Ang-2, MCP-1, IL-8, IL-6, PlGF, and VEGF-A were correlated with partial ischemic area in NPR and MPR. Levels of Ang-1 and IL-6 showed correlation with partial NPA only in the MPR. The correlations remained similar when assessed by ISI (Table 4). However, complete ischemic NPA and ISI for the entire retina and for each prespecified zone showed no correlation with any of the parameters including BCVA, CMT, and cytokine levels (Table 5).

**Table 4:** Correlation of partial ischemia in different retinal zones with visual acuity, central macular thickness, and levels of cytokines.

	BCVA		CMT		Ang-1		Ang-2		MCP-1		IL-8		IL-6		PDGF-AA		PlGF		VEGF-A	
	R	*P*	R	*P*	R	*P*	R	*P*	R	*P*	R	*P*	R	*P*	R	*P*	R	*P*	R	*P*
NPA	− 0.031	0.866	0.211	0.231	0.500	0.002*	0.344	0.043*	0.383	0.023*	0.416	0.013*	0.462	0.005*	0.106	0.545	0.358	0.035*	0.416	0.013*
PMR	0.171	0.348	0.440	0.009*	− 0.035	0.841	0.060	0.733	0.056	0.748	0.085	0.626	− 0.096	0.584	0.199	0.252	0.109	0.534	− 0.034	0.844
NPR	0.093	0.612	0.398	0.020*	0.224	0.195	0.420	0.012*	0.347	0.041*	0.371	0.028*	0.302	0.078	− 0.017	0.925	0.364	0.032*	0.404	0.016*
MPR	− 0.019	0.918	0.104	0.557	0.505	0.002*	0.348	0.041*	0.458	0.006*	0.412	0.014*	0.526	0.001*	0.201	0.248	0.342	0.044*	0.398	0.018*
FPR	− 0.288	0.109	− 0.241	0.170	0.265	0.123	0.114	0.515	0.113	0.518	0.086	0.623	0.200	0.248	0.131	0.452	0.051	0.771	0.178	0.307
ISI	− 0.036	0.844	0.252	0.150	0.389	0.021*	0.357	0.035*	0.383	0.023*	0.506	0.002*	0.439	0.008*	0.182	0.294	0.432	0.010*	0.444	0.008*
PMR	0.158	0.387	0.411	0.016*	− 0.003	0.987	0.166	0.339	0.116	0.507	0.103	0.555	− 0.027	0.878	0.199	0.252	0.154	0.378	0.002	0.990
NPR	0.083	0.652	0.444	0.009*	0.219	0.206	0.421	0.012*	0.352	0.038*	0.441	0.008*	0.337	0.047*	0.005	0.976	0.393	0.020*	0.426	0.011*
MPR	− 0.031	0.866	0.156	0.378	0.441	0.008*	0.338	0.047*	0.445	0.007*	0.467	0.005*	0.530	0.001*	0.272	0.113	0.359	0.034*	0.382	0.024*
FPR	− 0.281	0.119	− 0.190	0.282	0.105	0.549	0.074	0.674	0.144	0.410	0.160	0.358	0.160	0.358	0.233	0.178	0.113	0.519	0.195	0.262

BCVA: best-corrected visual acuity, CMT: central macular thickness, FPR: far-peripheral region; ISI: ischemic index, MPR: mid-peripheral region, NPA: non-perfusion area, NPR: near-peripheral region, PMR: peri-macular region, R: Spearman’s correlation coefficient.

*P*-values were calculated using Spearman’s correlation.

*P-value < 0.05.

**Table 5:** Correlation of complete ischemia in different retinal zones with visual acuity, central macular thickness, and levels of cytokines.

	BCVA		CMT		Ang-1		Ang-2		MCP-1		IL-8		IL-6		PDGF-AA		PlGF		VEGF-A	
	R	*P*	R	*P*	R	*P*	R	*P*	R	*P*	R	*P*	R	*P*	R	*P*	R	*P*	R	*P*
NPA	0.085	0.643	0.174	0.324	− 0.074	0.674	− 0.022	0.900	− 0.030	0.865	0.035	0.843	− 0.217	0.211	0.021	0.906	0.061	0.730	0.035	0.841
PMR	0.102	0.579	− 0.049	0.782	0.140	0.421	− 0.173	0.320	0.016	0.929	− 0.002	0.992	0.044	0.803	0.192	0.269	0.051	0.770	0.070	0.691
NPR	0.061	0.739	0.037	0.836	− 0.056	0.751	− 0.016	0.927	− 0.023	0.898	0.099	0.570	− 0.141	0.420	0.025	0.888	0.175	0.315	0.066	0.708
MPR	0.015	0.935	0.176	0.321	− 0.096	0.584	0.080	0.648	0.010	0.953	0.108	0.536	− 0.158	0.366	− 0.009	0.960	0.092	0.601	0.041	0.816
FPR	− 0.005	0.979	0.146	0.409	0.077	0.661	− 0.205	0.237	− 0.245	0.155	− 0.192	0.268	− 0.288	0.093	− 0.095	0.589	− 0.282	0.100	− 0.222	0.200
ISI	0.133	0.467	0.130	0.464	− 0.135	0.438	− 0.024	0.893	− 0.023	0.894	− 0.018	0.917	− 0.258	0.134	− 0.012	0.944	0.057	0.746	0.011	0.950
PMR	0.178	0.329	0.004	0.982	0.178	0.305	− 0.128	0.463	0.061	0.728	0.033	0.853	0.061	0.728	0.214	0.218	0.105	0.547	0.102	0.562
NPR	0.064	0.728	0.052	0.770	− 0.050	0.775	0.036	0.837	0.011	0.948	0.136	0.437	− 0.090	0.608	0.028	0.874	0.218	0.209	0.095	0.589
MPR	0.036	0.846	0.093	0.602	− 0.141	0.420	0.096	0.585	0.026	0.880	0.049	0.781	− 0.209	0.228	− 0.059	0.735	0.086	0.621	0.018	0.918
FPR	0.056	0.760	0.167	0.346	− 0.095	0.587	− 0.088	0.616	− 0.078	0.657	− 0.098	0.574	− 0.288	0.094	− 0.009	0.960	− 0.082	0.641	− 0.100	0.569

BCVA: best-corrected visual acuity, CMT: central macular thickness, FPR: far-peripheral region; ISI: ischemic index, MPR: mid-peripheral region, NPA: non-perfusion area, NPR: near-peripheral region, PMR: peri-macular region, R: Spearman’s correlation coefficient.

*P*-values were calculated using Spearman’s correlation.

*P-value < 0.05.

### Correlation of cytokine levels with clinical parameters of BRVO

There was no significant correlation between cytokine levels and baseline BCVA. However, the levels of Ang-2 (r = 0.442, *P* = 0.009), MCP-1 (r = 0.356, *P* = 0.039), IL-8 (r = 0.394, *P* = 0.021), PlGF (r = 0.448, *P* = 0.008) and VEGF-A (r = 0.384, *P* = 0.025) showed significant correlation with CMT. Baseline BCVA and CMT showed a significant correlation (r = 0.641, *P* < 0.001).

### Correlation matrix of cytokines

In the BRVO group, significant correlations were noted between the levels of all cytokines except for Ang-1 and Ang-2 (Supplementary Table S1).

## Discussion

In the present study, we investigated the association between the levels of aqueous cytokines and the severity of retinal ischemia in patients with BRVO. As expected, the aqueous levels of angiogenic and inflammatory cytokines were higher in the BRVO group than the control group. The distribution of ischemic areas (as quantified by ISI) in the BRVO group showed significant differences among the retinal zones, with the greatest percentage of ISI observed in the PMR. The proportion of complete ischemic to total ischemia was the highest in the FPR. The proportion of partial ischemia to total ischemia, however, was higher in the posterior regions than the peripheral retina. This is important, as only the partial ischemia, particularly in regions posterior to the FPR, showed significant correlation with CMT and the aqueous levels of Ang-1, Ang-2, MCP-1, IL-8, IL-6, PlGF, and VEGF-A. Notably, partial ischemia in the far periphery and complete ischemia (in any region) did not show any correlation with these parameters. CMT showed significant correlation with the aqueous levels of Ang-2, MCP-1, IL-8, PlGF, and VEGF-A. This would seem to emphasize the importance of these other cytokines to the pathophysiology of macular edema in BRVO.

**Figure 1:**
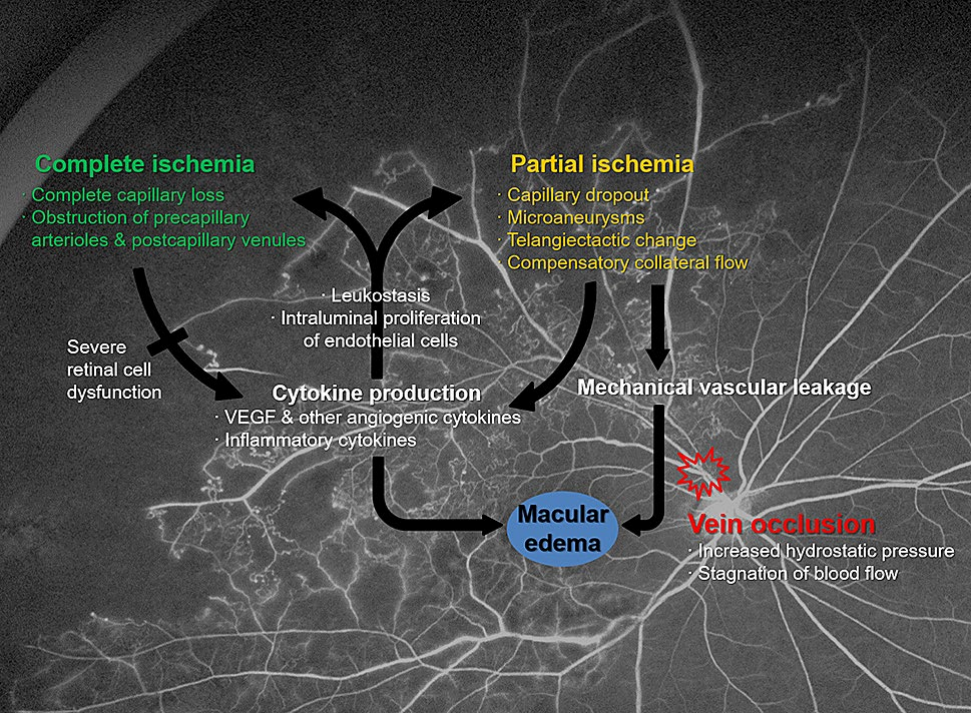
Schematic representation suggesting the relationship between retinal ischemia, cytokine production, and macular edema in patients with branch retinal vein occlusion. Vein occlusion is accompanied by increased hydrostatic pressure and blood flow stagnation, causing retinal ischemia, predominantly partial ischemia in peri-macular and near-peripheral region and complete ischemia in mid- and far-peripheral region. Partial ischemia may cause macular edema, in consequence of vascular hyperpermeability through increased cytokine secretion, such as VEGF, and direct leakage from damaged vessels. While, complete ischemia contributes less to cytokine production and direct leakage due to severe retinal cell dysfunction and loss of vessels. Increased cytokines can lead to complete ischemia progression through leukostasis and intraluminal proliferation of vascular endothelial cells within capillaries and venules.

There are conflicting opinions regarding the relationship between the status of retinal perfusion and ME in BRVO^7–9^. However, a previous study which used a definition of non-perfusion similar to that of the current study also reported that the areas of partial capillary loss (rather than complete capillary loss) are associated with ME secondary BRVO^10^. The authors suggested that with a greater extent of completely ischemic/ non-perfused retina, there is an overall decrease in the number of vessels, which are the ultimate source of leakage^10^. We would tend to agree with this formulation. With complete ischemia, there is an essentially an infarction of the inner retinal tissue. This means a loss of cells requiring oxygen (i.e. reduced demand) as well as a reduction in the number of cells producing cytokines in response to ischemia—and thus less edema^15^. In contrast, in regions of partial ischemia, the cells are presumably still alive and capable of producing cytokines in response to the ischemic insult. This cytokine elevation can in turn lead to increased vasopermeability and edema development (reflected in the increased CMT). Increased VEGF production, however, also causes leukostasis and intraluminal proliferation of vascular endothelial cells within capillaries and venules which can ultimately resulting in complete non-perfusion (Fig. 1)^16,17^ .Further study to evaluate the clinical significance of vascular leakage on cytokine level and macular thickness in BRVO should be conducted to confirm the current conclusions. It is notable that these regions of complete non-perfusion tended to be more extensive in the far-periphery. As the far periphery represents the terminal most extent of the retinal circulation, it is perhaps not surprising that these regions were susceptible to more severe or complete ischemia.

Of note, CMT and the levels of cytokines showed significant correlation with ischemia in the PMR and NPR. Kwon et al.^18^ also demonstrated that in patients with RVO and recurrent ME, the severity of ME was correlated only with ISI of PMR and the reduction in ME was correlated with the reduction in ISI in PMR and NPR following targeted retinal photocoagulation (TRP). Taken together, both our study and that of Kwon et al. would appear to support the overall contention that ischemia closer to the macula (and not the far periphery) is most relevant to cytokine production and development of ME^19,20^. The results from the RELATE (Ranibizumab Dose Comparison and the Role of Laser in Retinal Vein Occlusions; NCT01003106) and the WAVE (Wide-field Angiography Guided Targeted Retinal Photocoagulation Combined with Anti-VEGF Intravitreal Injections for the Treatment of Ischemic Retinal Vein Occlusion; NCT01710839) trials also highlighted that peripheral photocoagulation does not appreciably affect the treatment burden or visual outcomes in RVO patients^14,19,20^. The TRP in these trials was primarily aimed at the far-peripheral retina and presumably to areas of more complete ischemia – regions which may be less relevant to the presumed pathophysiology of ME in these eyes. One wonders whether different results would have been observed in these studies if the laser was targeted to more posterior regions of partial ischemia. Since the density of photoreceptors with high metabolic demands is low in the periphery, it is considered to be less susceptible to hypoxic damage than posterior retina. Additionally, it is thought that the physiologic peripheral nonperfused area located near the ora serrata may have influenced the results.

The present study also demonstrated that the levels of angiogenic and inflammatory cytokines were higher in aqueous humor from the BRVO group when compared with the control group. Similar to previous studies^2,3,5^, we also found that the increase in cytokine levels were correlated with the severity of ME and most of the cytokines were significantly correlated with each other in patients with BRVO. In particular, the aqueous levels of Ang-2, MCP-1, IL-8, PlGF, and VEGF-A showed significant correlation with CMT. In the presence of VEGF-A, Ang-2 promotes angiogenesis and vascular sprouting and MCP-1promotes chemotaxis and adhesion of leukocytes along with a decrease of local blood flow velocity and create positive feedback loop, which further promotes retinal hypoxia^6,21^. IL-8 promotes the upregulation of VEGF and links the inflammatory process with angiogenesis^2^. PlGF is also a member of the VEGF family and leads to releasing angiogenic factors and inflammatory reactions. Besides anti-VEGF agents, new therapeutic strategies targeting other angiogenic or inflammatory cytokines can be developed for the treatment of ME in BRVO.

Our study has several limitations which should be considered when assessing our findings. Due to the sample size, we could not perform a multivariate regression analysis to assess factors affecting CMT or BCVA in patients with BRVO. As we did not perform follow-up UWF FA and did not collect follow-up aqueous samples from the patients, we are unable to assess for longitudinal changes in retinal non-perfusion or cytokine levels. In addition, as the measurements of ischemia were based on manual segmentation, they were subject to potential inter-observer variability. To mitigate this limitation, the average values from two independent masked ophthalmologists were used and both graders followed a standardized grading protocol with pre-specified definitions of partial and complete ischemic areas. The Kappa values between graders with an average of 0.91 (range, 0.88 to 0.94) emphasizes a high-level of repeatability. A final limitation is that the aqueous cytokine levels may not reflect the actual findings in the retina, since cytokines in the aqueous humor are affected by their diffusion rate and their binding to extracellular matrix. However, obtaining vitreous (rather than aqueous) carries with it a greater risk (which would be especially difficult to justify in the controls), and previous studies have reported significant correlations among cytokine levels in vitreous and aqueous humor^4,5^. Plasma sample analysis was also not performed in this study because intraocular cytokines are supposed to come from an intraocular source rather than from the systemic circulation^22,23^. Nonetheless, it is the first study to investigate the clinical parameters according to regional distribution and severity based on metric unit from the UWF angiography. Moreover, we would note that our study also has several strengths including its prospective design, use of multiple graders, and a standardized grading protocol.

In summary, we observed that the extent of partial retinal non-perfusion in the more posterior retina (and not the far periphery) was correlated with various angiogenic and inflammatory cytokines and the severity of macular edema in eyes with BRVO. These findings may be of relevance to the design (e.g. Ang-2 inhibitors) and targeting (e.g. laser photocoagulation) of future therapeutics of BRVO.

## Methods

This prospective cohort study was conducted at the Retina Clinic of Yeungnam University Hospital in Daegu, South Korea. The Institutional Review Board of Yeungnam University Medical Center approved the study. The study was carried out in accordance with the Declaration of Helsinki, and written informed consent was obtained from all the patients.

### Participants

Thirty-five consecutive patients with treatment-naïve ME due to major BRVO were included. To be included, patients had to have clinically detectable symptomatic ME with CMT of 300 µm or greater on spectral-domain optical coherence tomography (SD-OCT) and occlusion of major branch retinal veins that affects the entire sector of the retina up to the periphery in the study eye. Exclusion criteria included: (a) poor image quality which precluded evaluation of non-perfusion, (b) previous treatment for ME including anti-VEGF therapy, laser photocoagulation, or vitrectomy, (c) a history of ocular surgery within 6 months, (d) diabetes mellitus or uncontrolled hypertension, and/or (e) a history of ocular inflammation or other vitreoretinal diseases.

Twenty-two age-matched control subjects without any history of systemic or ocular diseases, who were scheduled to undergo cataract surgery, were included in the study.

### Collection and analysis of cytokines

Before performing intravitreal anti-VEGF injection or cataract surgery (in the case of controls), a mean volume of 0.1 mL of aqueous humor was collected by anterior chamber paracentesis with a 30-gauge needle in sterile tubes. The samples were immediately transferred to a sterile plastic tube and frozen at − 80 °C until analysis.

The cytokine levels were measured using multiplex bead analysis (xMAP, Luminex Corp., Austin, TX, USA). Capture bead kits (Beadlyte, Upstate Biotechnology, Lake Placid, NY, USA) were used for the detection of Ang-1, Ang-2, MCP-1, IL-6, IL-8, PDGF-AA, PlGF, and VEGF-A. These particular cytokines were pre-specified as they are known to be angiogenic or pro-inflammatory, and have been suggested to be relevant to macular edema^2–6^. The concentrations of cytokines were calculated in duplicate from the standard curves of each cytokine tested using the Master Plex QT 2010 software (Miraibio, Hitachi, CA, USA).

### Ophthalmic examinations

All patients underwent complete ophthalmic examination at the initial visit including best-corrected visual acuity (BCVA) and dilated ophthalmoscopy. BCVA was measured using a Snellen chart and converted to logMAR values for analysis. In addition, the subjects with BRVO underwent SD-OCT (Spectralis, Heidelberg Engineering, Heidelberg, Germany) and UWF FA (Optos California, Optos plc, Dunfermline, UK) before intravitreal anti-VEGF injection. To avoid confusion between NP and blocked fluorescence, angiographic evaluation was repeated after regions of thick hemorrhage were resorbed. The presence and extent of non-perfusion was assessed in the late transit phase of the angiogram (about 45 s after the intravenous injection of fluorescein dye).

### Measurement of NPA and ISI using UWF FA

UWF FA images were transformed into stereographic projection images using the manufacturer’s software. The projection was performed by ray-tracing every pixel through a combined optical model with an axial length of 24 mm, thus transforming the 3-D retinal surface to a 2-D image.

**Figure 2:**
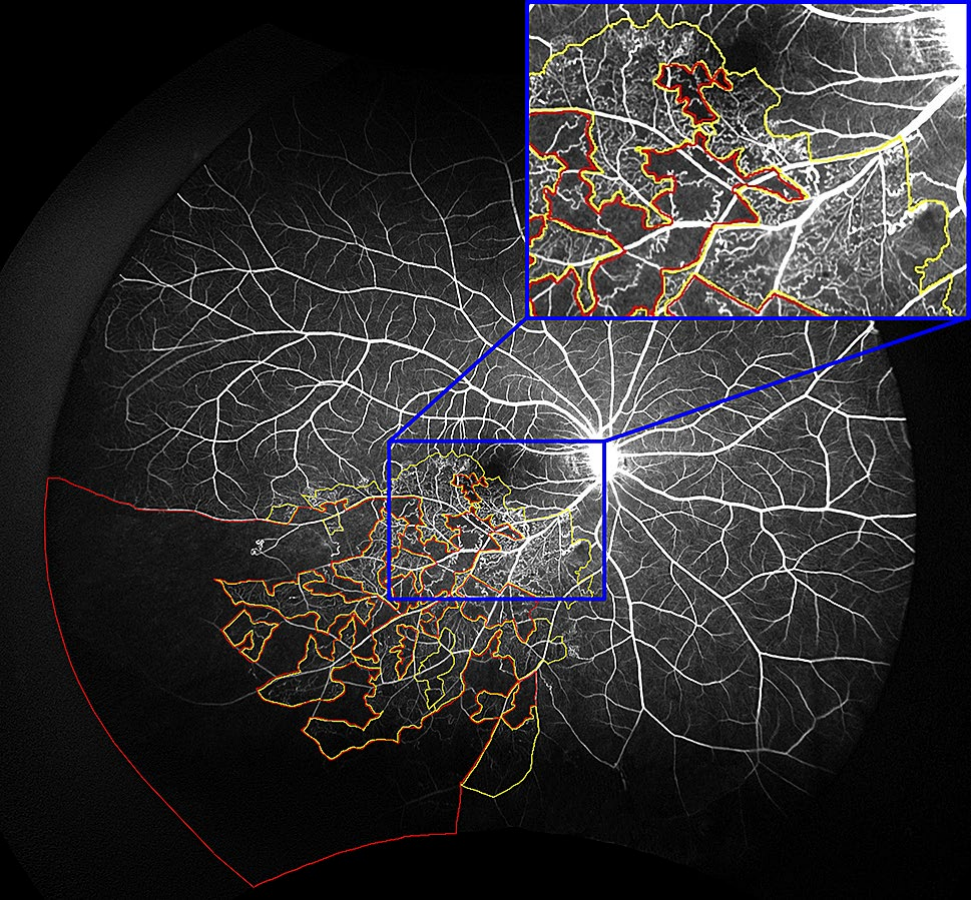
Ultra-widefield fluorescein angiogram of branch retinal vein occlusion with various non-perfusion status. Areas of partial ischemia (surrounded by yellow lines) show clusters of small fragmented areas of hypofluorescence with dilation and irregular capillary network patterns. Areas of complete ischemia (surrounded by red lines) show continuous area of hypofluorescence due to retinal capillary loss and obstruction of precapillary arterioles and postcapillary venules.

The regions of non-perfusion were subclassified as partially ischemic or completely ischemic, using similar criteria as in previous publications^10^. A partial ischemic area was defined as the presence of a cluster of small fragmented areas of hypofluorescence with dilation and irregular patterns of the capillary network (Fig. 2). A complete ischemic area was defined as a contiguous area of hypofluorescence due to retinal capillary loss and the obstruction of the precapillary arterioles and postcapillary venules, with the remaining precapillary arterioles and postcapillary venules dilated and tortuous (Fig. 2).

Two masked retinal specialists (D.N. and G.R.) independently analyzed the images. For all analyses, the average values from the two independent graders were used. Using ImageJ version 1.51j8 (National Institute of Health, Bethesda, MD, USA), graders manually outlined the border of the NPA and the peripheral extent of the total visible retinal area (TRA). Grading results were exported as a binary mask and were automatically calculated in square millimeters (mm^2^) by summing the size of all the pixels of the mask using manufacturer’s quantification software. The size of a pixel was defined by its location in the macula-centered image. It was calculated using spherical trigonometry after reversing the projection onto a 3-dimensional representation^24^. ISI was calculated by dividing the NPA by the TRA.

To assess the amount and severity of non-perfusion in different retinal regions, we created a grid with several concentric rings centered on the fovea to define four zones: PMR (0.5–3 mm radius), NPR (3–10 mm), MPR (10–15 mm), and FPR (15 mm-normal perfusion boundary)^25^. The central 1 mm diameter including the foveal avascular zone was masked and excluded from the evaluation of non-perfusion.

### Statistical analysis

Statistical analysis was performed using SPSS 20.0 for windows (SPSS Inc., Chicago, IL, USA). Mann–Whitney U test was used to compare the continuous variables and Chi square test was used to compare frequency data between the controls and the BRVO patients. NPA and ISI within the 4 zones were compared using the Friedman test. The correlation of NPA and ISI from the prespecified zones with cytokine concentrations, BCVA, and CMT was calculated using Spearman’s rank correlation. The same test was also used to examine the relationships among the cytokines. Two-tailed *P*-values of less than 0.05 were considered statistically significant. Kappa statistics were used to assess the level of agreement between the two graders.

## Supplementary information

Supplementary Information 1.

## Data Availability

The datasets generated during and/or analysed during the current study are available from the corresponding author on reasonable request.
