# Supplementary material for: Relationship between distribution and severity of non-perfusion and cytokine levels and macular thickness in branch retinal vein occlusion
Source: Sci Rep. 2021 Jan 11;11:271. doi: 10.1038/s41598-020-79522-5 (PMC7801443; doi:10.1038/s41598-020-79522-5)
Supplement: Supplementary file 1 — Supplementary Information 1. [file 41598_2020_79522_MOESM1_ESM.pdf]

Relationship between distribution and severity of non-perfusion and cytokine levels and macular thickness in branch retinal vein occlusion

Gahyung Ryu[1,2], Donghyoun Noh[1,3], Jano van Hemert[4], SriniVas R. Sadda[5,6], Min Sagong[1,2*]

[1]Department of Ophthalmology, Yeungnam University College of Medicine, Daegu, South Korea

[2]Yeungnam Eye Center, Yeungnam University Hospital, Daegu, South Korea

[3]Good Doctors Eye Hospital, Ulsan, South Korea

[4]Optos PLC, Dunfermline, United Kingdom

[5]Doheny Image Reading Center, Doheny Eye Institute, Los Angeles, CA, USA

[6]Department of Ophthalmology, David Geffen School of Medicine at UCLA, Los Angeles, CA, USA

***Corresponding Author:** msagong@ynu.ac.kr

Supplementary Table S1. Correlation matrix of cytokines

	Ang-1		Ang-2		MCP-1		IL-8		IL-6		PDGF-AA		PlGF		VEGF-A	
	R	*P*	R	*P*	R	*P*	R	*P*	R	*P*	R	*P*	R	*P*	R	*P*
Ang-1			0.304	0.076	0.589	<0.001*	0.405	0.016*	0.643	<0.001*	0.541	0.001*	0.373	0.027*	0.432	0.009*
Ang-2					0.688	<0.001*	0.774	<0.001*	0.557	0.001*	0.386	0.022*	0.790	<0.001*	0.777	<0.001*
MCP-1							0.798	<0.001*	0.854	<0.001*	0.585	<0.001*	0.715	<0.001*	0.776	<0.001*
IL-8									0.778	<0.001*	0.407	0.015*	0.825	<0.001*	0.797	<0.001*
IL-6											0.500	0.002*	0.582	<0.001*	0.712	<0.001*
PDGF-AA													0.463	0.005*	0.469	0.004*
PlGF															0.806	<0.001*

R: Spearman correlation coefficient

P-values were calculated using Spearman rank correlation.

**P*-value < 0.05
